# Simulated Microgravity Inhibited the Proliferation of Bovine Muscle Satellite Cells

**DOI:** 10.33549/physiolres.935732

**Published:** 2026-08-01

**Authors:** Nguyen Quynh Chi HO, Thai Minh NGUYEN, Minh Thuy DOAN, Bao Khoi NGUYEN, Minh Anh PHAM, Minh Cuong LE PHAN, Lu Chinh Nhan PHAN, Thi Tung Loan DANG, Huu Trinh LE, Chinh Chung DOAN, Nghia Son HOANG, Thai Minh Han NGUYEN, Quang Manh VU, Thanh Long LE

**Affiliations:** 1 Biotechnology Department, Graduate University of Science and Technology, Vietnam Academy of Science and Technology, Ha Noi, Vietnam; 2 Animal Biotechnology Department, Institute of Life Sciences, Vietnam Academy of Science and Technology, Ho Chi Minh City, Vietnam; 3 Faculty of Biology and Biotechnology, University of Science, Vietnam National University, Ho Chi Minh, Vietnam; 4 Center for Biotechnology and Genomic Medicine, Medical College of Georgia, Augusta University, Augusta, USA; 5 Life Science Department, University of New Hampshire at Manchester, Manchester, USA; 6 Institute of Life Science and Application, Hoa Binh University, Bui Xuan Phai Street, Hanoi, Vietnam

**Keywords:** Cell cycle, Proliferation, Satellite cells, Simulated microgravity

## Abstract

This study aimed to assess the effects of simulated microgravity (SMG) on bovine muscle satellite cells (bSCs). The results demonstrated that bSCs maintained a fibroblast-like morphology throughout SMG exposure from day 3 to day 7. However, cells in the SMG group exhibited reduced proliferation compared to the control group, as indicated by lower cell density at both time points. In contrast, cell viability was higher in the SMG group than in the control group. Cell cycle analysis revealed a higher proportion of SMG-treated bSCs in the G0/G1 phase and a lower proportion in the S phase at day 3, suggesting cell cycle arrest at an early stage. Interestingly, by day 7, the percentage of cells in the S phase increased in the SMG group and exceeded that of the control group, indicating a partial recovery of proliferative activity. Western blot analysis showed decreased expression of key cell cycle-related proteins, including cyclin A1/A2, cyclin D1, and CDK2, in SMG-treated cells compared to controls at the early stage. Notably, cyclin D1 and CDK2 expression levels were upregulated at the later stage under SMG conditions. Additionally, nuclear morphology in SMG-treated bSCs displayed enlargement and irregularity. Overall, these findings suggest that SMG initially suppresses bSC proliferation by inducing cell cycle arrest, but cells can partially retrieve their proliferative capacity at later stages, potentially through upregulation of cell cycle regulatory proteins.

## Introduction

Long-duration space missions, whether aboard spacecraft or at planetary outposts such as the Moon or Mars, will require sustainable *in situ* food production systems. This approach can reduce reliance on transported long-shelf-life provisions, ensure a more stable and diversified nutritional supply, mitigate risks associated with food degradation or loss, enhance mission self-sufficiency, and provide access to fresh food sources. Cellular agriculture offers a promising solution by enabling the production of substantial quantities of cultured meat from a relatively small population of animal muscle cells [1]. Notably, in 2019, preliminary success was achieved in generating small-scale cultured meat aboard a space station [2]. Nevertheless, the development of artificial meat production in space remains constrained by significant challenges, particularly the limited understanding of muscle cell proliferation and adaptive responses under microgravity and spaceflight conditions, which necessitates further investigation.

Cell proliferation is significantly influenced by microgravity, as both real and simulated conditions alter the physical and biochemical environments of cells. In mammalian systems, microgravity generally suppresses proliferation by disrupting cytoskeletal organization, weakening focal adhesions, and impairing mechanotransduction. These changes can induce G0/G1 cell cycle arrest and increase markers of cellular senescence and apoptosis [3–4]. However, some studies report enhanced proliferation or survival in certain cell types under microgravity, potentially due to dysregulation of apoptotic pathways and activation of oncogenic signaling [5–6]. Key signaling pathways, including PI3K/Akt, MAPK/ERK, and YAP/TAZ, which are normally responsive to mechanical cues, are particularly sensitive to gravitational unloading, resulting in widespread alterations in cell proliferation, migration, and stemness [7–8].

Furthermore, studies on muscle satellite cells have demonstrated reduced proliferative capacity and altered differentiation potential under microgravity conditions, raising concerns about musculoskeletal deterioration during long-term spaceflight. Collectively, these findings indicate that microgravity exerts cell type–specific effects, generally inhibiting normal somatic cell proliferation while potentially promoting malignant-like behaviors. Despite these advances, the long-term in vitro proliferative responses of satellite cells under microgravity remain poorly characterized. Therefore, this study aimed to investigate the effects of simulated microgravity, generated using the Gravite® 3D clinostat, on bovine satellite cells, with a focus on changes in cell density, viability, cell cycle progression, and cellular morphology.

## Materials and Methods

### Cell culture

Bovine satellite cells (bSCs) were maintained in DMEM/Ham’s F-12 medium (DMEM-12-A, Capricorn Scientific, Ebsdorfergrund, Germany) supplemented with 15% fetal bovine serum (FBS-HI-22B, Capricorn Scientific, Ebsdorfergrund, Germany) and 1% penicillin–streptomycin (15140-122, Gibco, Thermo Fisher Scientific, Waltham, MA, USA) [9]. For simulated microgravity (SMG) experiments, cells were seeded at a density of 1 × 10^3^ cells per well in 96-well plates. Each plate was gently filled with culture medium to eliminate bubbles and minimize fluid shear stress [10]. The plates were then mounted onto the inner frame of a 3D clinostat (MiGra-ITB, Vietnam Academy of Science and Technology, Ha Noi, Vietnam) and placed in a CO_2_ incubator (Sanyo MCO-18AIC, Sanyo Electric Co., Osaka, Japan). Cells were cultured under SMG for seven days, while control cells were maintained under normal gravity (1 g) in the same incubator.

### Cell density measurement

bSCs were seeded at 1 × 10^3^ cells per well in 96-well plates (Thermo Fisher Scientific, USA). Wells were completely filled with culture medium and sealed with parafilm before exposure to SMG for seven days. After treatment, cultures were discarded and nuclei were stained with Hoechst 33342 (Sigma-Aldrich, Germany) for 15 minutes, followed by three PBS washes. Cell numbers were quantified using the Cell Cycle Application of a Cytell microscope (GE Healthcare, USA) [11].

### WST-1 cell viability assay

After seven days of SMG exposure, culture medium was replaced with 100 μL fresh medium containing 10 μL WST-1 reagent (11644807001, Roche, Basel, Switzerland) [12]. Cells were incubated for 3.5 h at 37 °C in a 5% CO_2_ atmosphere. Absorbance was measured at 450 nm using a GloMax Explorer microplate reader (Promega Corporation, Fitchburg, WI, USA). Parallel cultures maintained under 1 g served as controls.

### Microfilament staining and nuclear morphology evaluation

bSCs cultured under SMG for seven days were fixed with 4% paraformaldehyde (Nacalai Tesque, Kyoto, Japan) for 30 minutes, then permeabilized overnight with 0.1% Triton X-100 (Merck, Darmstadt, Germany) at 4 °C. Actin filaments were stained with phalloidin CruzFluor™ 488 (sc-363791, Santa Cruz Biotechnology, Santa Cruz, CA, USA) for 1 h, while nuclei were counterstained with Hoechst 33342 (14533, Sigma-Aldrich, Munich, Germany) for 15 minutes. After three PBS washes (Gibco, Thermo Fisher Scientific, Inc., Waltham, MA, USA), samples were imaged using a Cytell microscope. Nuclear area, intensity, and shape parameters were quantified with the Cell Cycle Application [13].

### Western blot

Cells were lysed using Optiblot LDS Sample Buffer (ab119196, Abcam, Cambridge, MA, USA). Equal amounts of protein were loaded onto 4–12% SDS–PAGE gels and separated by electrophoresis using running buffer (ab119197, Abcam) at 50 V for 2 h. The separated proteins were then transferred onto a methanol-activated PVDF membrane (ab133411, Abcam). Membranes were blocked overnight at 4 °C using blocking buffer (ab126587, Abcam). Following blocking, membranes were incubated overnight at 4 °C with primary antibodies. Anti-cyclin A1/A2 (ab185619, Abcam), Anti-Cyclin D1 (ab40754, Abcam), and anti-CDK2 (ab32147, Abcam) antibodies were also applied at 1:5000 dilutions. Anti-GAPDH antibody (ab181602) was used as a loading control at a dilution of 1:10,000. After primary antibody incubation, membranes were washed with TBST and incubated with HRP-conjugated goat anti-rabbit IgG secondary antibody (ab6721, Abcam) for 1 h at room temperature. Protein bands were visualized using an enhanced chemiluminescence substrate kit (ab65623, Abcam) and detected on X-ray film [14].

### Statistical analysis

All experiments were performed in triplicate. Data are expressed as mean ± standard deviation. Statistical significance was evaluated by one-way ANOVA, with *p* < 0.05 considered significant.

## Results

### Proliferation and availability of satellite cells under SMG

**Fig. 1:**
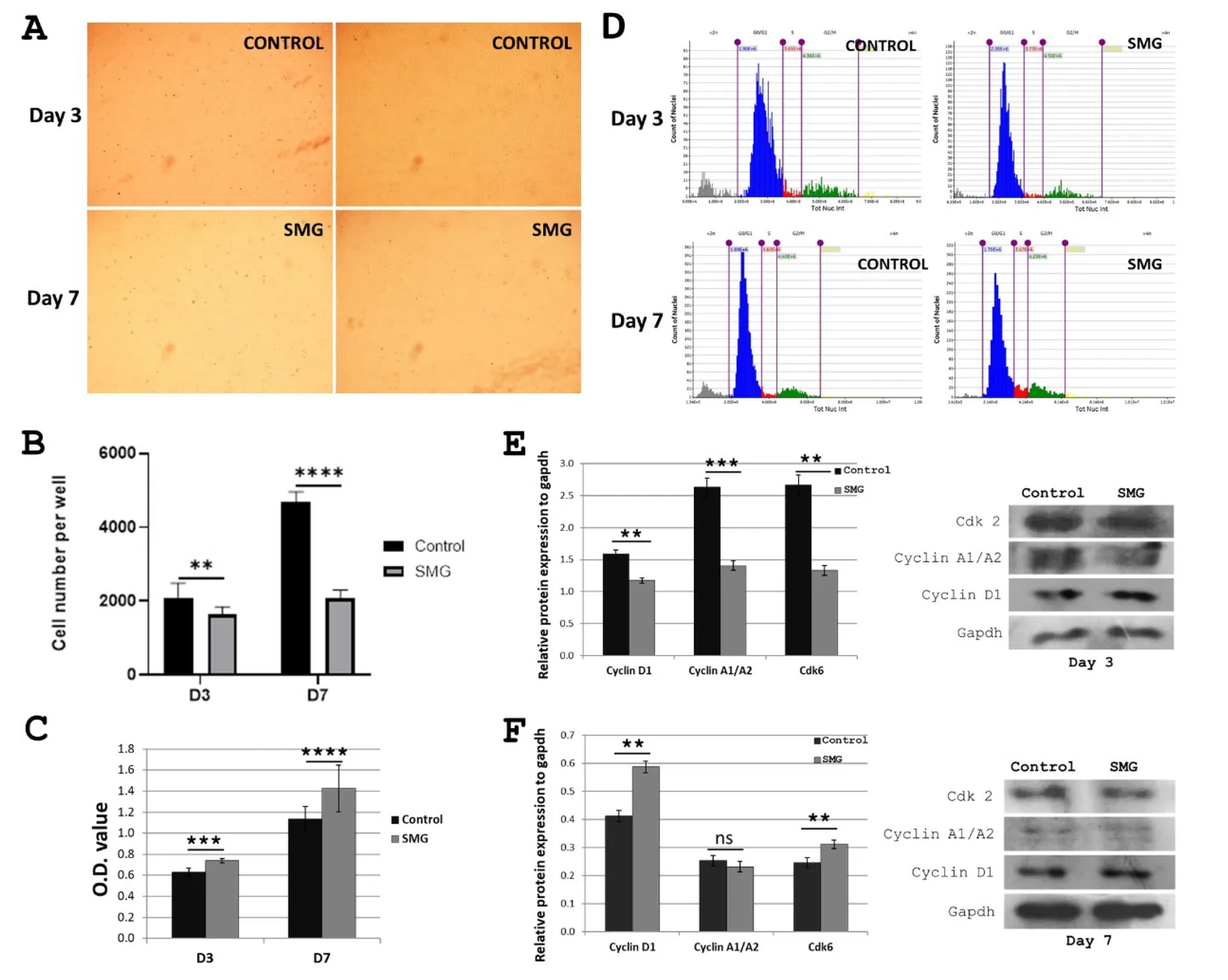
bSC proliferation. **A**) Cell morphology from control and SMG groups. **B**) The number of bSCs was counted using the Cell Cycle App. of the Cytell microscope. **C**) Cell viability was assessed by WST-1 assay. **D**) Cell cycle analysis was performed by Cell Cycle App. of the Cytell microscope. **E**) Protein expression of bSCs at day 3 analyzed by western blot. **F**) Protein expression of bSCs at day 7 analyzed by western blot. ** indicates significant difference compared with the control group (p < 0.01). *** indicates significant difference compared with the control group (p < 0.001). **** indicates significant difference compared with the control group (p < 0.0001).

Fig. 1A illustrated the proliferation of bSCs from control group and SMG group, demonstrating by the fibroblast-like shape with an elongated, spindle-shaped morphology. The number of satellite cells was determined using Cell cycle App. of the Cytell Microscope (GE Healthcare Electronics, USA). The cell number in the control group was significantly higher than the SMG group both in day 3 and day 7 (Fig. 1B). The cell density in the control group was increased from day 3 (2.1 × 10^3^ cells/well) to day 7 (4.7 × 10^3^ cells/well). bSCs from SMG group showed a lower proliferation from day 3 (1.6 × 10^3^ cells/well) to day 7 (2.1 × 10^3^ cells/well). The Wst-1 assay showed that the O.D. value of the control group was lower than the SMG group both in day 3 and day 7 (Fig. 1C). The O.D. value of bSCs from control group increase from day 3 to day 7 (0.63 ± 0.04 and 1.14 ± 0.12, respectively). The O.D. value of bSCs from SMG group increase from day 3 to day 7 (0.74 ± 0.02 and 1.43 ± 0.22, respectively).

The changes in cell cycle progression were demonstrated in Fig. 1D and Table 1. At day 3, the G0/G1 percentage of bSCs from control group was lower than SMG group (73.34 ± 2.13 % vs 80.51 ± 1.77 %, P < 0.001), while the S percentage of bSCs from control group was higher than SMG group (4.88 ± 1.23 % vs 2.34 ± 0.39 %, P < 0.01). An increase in G0/G1 percentage of bSCs from control group (77.71 ± 3.09 %) was observed at day 7. Notably, the S percentage of bSCs from SMG was increased and higher than control group at day 7 (7.92 ± 1.78 % vs 3.64 ± 2.37 %). There was no significant difference in G2/M between control group and SMG group. The cell percentage in sub G1 from the control group was higher than the SMG at day 3 and day 7. The sub G1 percentage of bSCs from control group was increased from day 3 to day 7, while it was decreased in the SMG group.

In this study, western blotting was employed to evaluate the expression of cell cycle related proteins. As shown in Fig. 1E and Fig. 1F, bSCs from control group exhibited higher expression of cyclin A1/A2, cyclin D1 and cdk6 compared to the SMG group at day 3. At day 7, a down-regulation was observed in these proteins in both groups compared to day 3. Interestingly, we found that the expression of cyclin D1 and cdk6 of bSCs in SMG group were higher than the control group at day 7.

**Fig. 2:**
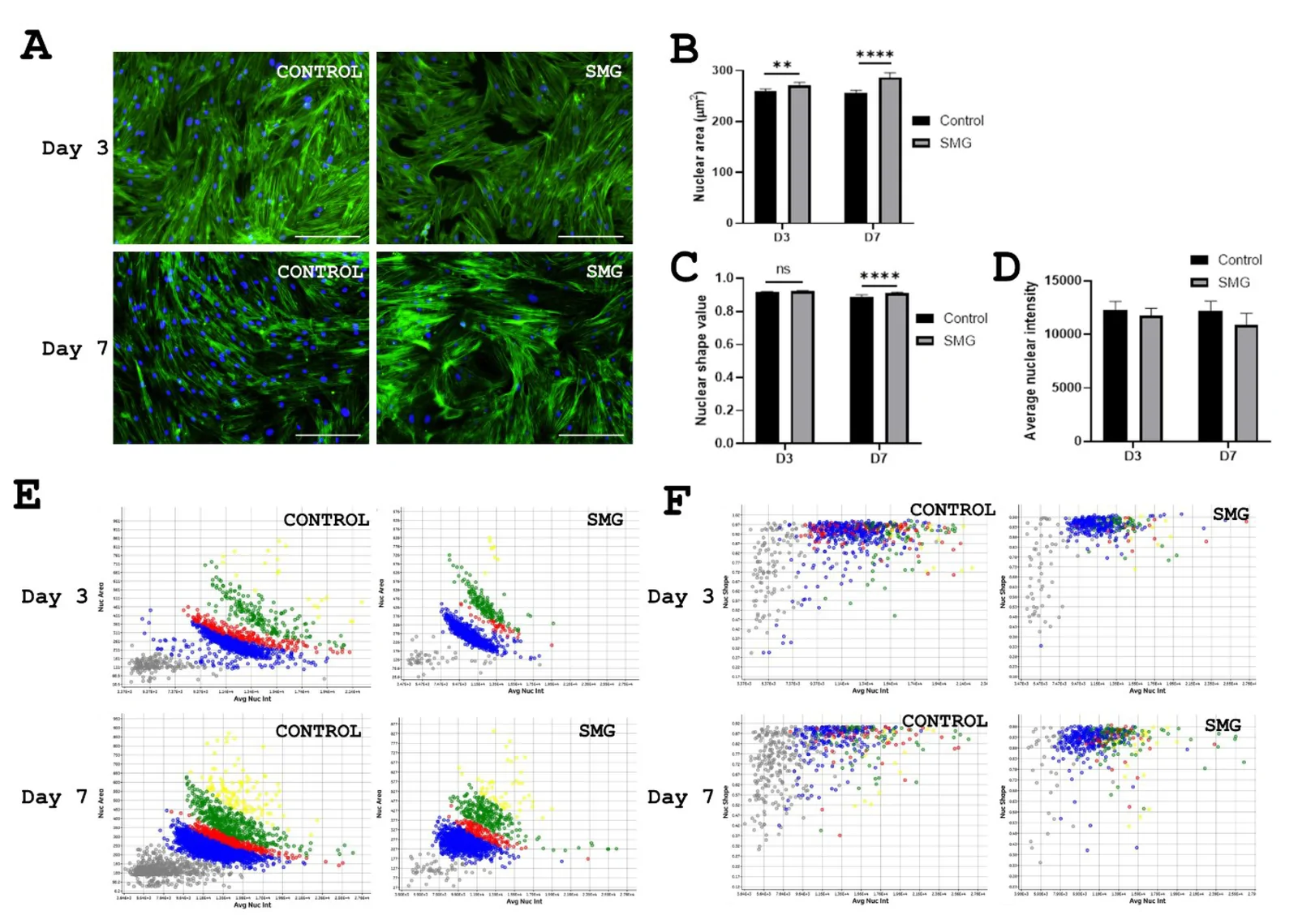
The changes in bSC morphology. **A**) Distribution of microfilament bundles in bSCs, nuclei were counterstained with H33342 (blue), and microfilaments were counterstained with Phalloidin (green), scale bar = 223.64 μm. **B–D**) Nuclear area, Nuclear shape value, Average nuclear intensity (respectively). ** indicates significant difference compared with the control group (p < 0.01). **** indicates significant difference compared with the control group (p < 0.0001). **E**) The distribution of the nuclei for both the nuclear-area value and the total nuclear-intensity value. **F**) The distribution of the nuclei for both the nuclear-shape value and the total nuclear-intensity value.

Fig. 2A demonstrated the nuclear morphology of bSCs in the control group and SMG group. The nuclear area of the control group was lower than the SMG group in both day 3 and day 7. There was no significant difference in nuclear area of the control between day 3 and day 7 (260.1 ± 3.1 μm^2^ and 255.8 ± 5.9 μm^2^, respectively), while an increase was observed in the SMG group (270.1 ± 6.7 μm^2^ and 285.8 ± 9.7 μm^2^, respectively) (Fig. 2B and Fig. 2E).

The nuclear shape value showed no significant difference between groups at day 3. However, by day 7, the control group exhibited a lower nuclear shape value compared to the SMG group. Specifically, in the control group, the nuclear shape value decreased from 0.92 ± 0.01 at day 3 to 0.89 ± 0.01 at day 7, whereas in the SMG group, it decreased more modestly from 0.92 ± 0.01 to 0.91 ± 0.01 (Fig. 2C and Fig. 2F).

The average nuclear intensity was no significant difference between groups in day 3, but the control group was higher than SMG group in day 7. The average nuclear intensity in the control group was no significant change (12262 ± 802 and 12259 ± 869, respectively), while the SMG group decreased form 11738 ± 674 in day 3 to 10865 ± 1099 in day 7 (Fig. 2D).

## Discussion

Accumulating evidence indicates that disruption of cytoskeleton–nucleus coupling and impaired mechanotransduction signaling are key drivers of altered proliferation in muscle cells exposed to SMG. In both primary myogenic cells and C2C12 cells—an established immortalized mouse myoblast model—SMG consistently reduces BrdU/EdU incorporation, delays myogenic differentiation, and weakens responsiveness to growth factors, with effects predominantly mapped to the G1/S checkpoint [15–16]. Furthermore, SMG has been reported to induce marked nuclear remodeling, including nuclear enlargement, folding, and blebbing, accompanied by chromatin decondensation and reorganization of lamin and actin networks, all of which correlate with cell-cycle delay and cellular stress phenotypes [17–18].

In agreement with these reports, our study shows that SMG significantly inhibits bovine satellite cell (bSC) expansion at both examined time points, with proliferation reaching only 1.3-fold compared to 2.2-fold in 1g controls. This reduced proliferative capacity is accompanied by pronounced nuclear remodeling, as evidenced by increased nuclear size, enhanced shape irregularity, and decreased nuclear intensity. These structural alterations are indicative of reduced DNA replication activity (lower S-phase fraction) and decreased chromatin condensation [19], both of which are hallmarks of cell-cycle arrest. Mechanistically, such changes may arise from impaired force transmission between the cytoskeleton and nucleus, leading to altered nuclear architecture and transcriptional regulation.

In this study, a higher proportion of cells in the sub-G1 phase was observed in the control group compared to the SMG group. The sub-G1 population corresponds to early apoptotic cells, which exhibit DNA fragmentation and hypodiploid DNA content, a reliable apoptotic indicator [20,21]. In flow cytometry analysis, these apoptotic cells form a distinct hypodiploid peak that appears before the G0/G1 peak in the DNA content histogram. The presence of this peak is widely used to identify apoptotic events, and the percentage of sub-G1 cells relative to the total cell population is commonly used to quantify the extent of apoptosis [22]. Notably, in contrast to the reduced proliferation observed in bSCs, bGCs cultured under SMG exhibited increased WST-1 absorbance despite lower cell numbers. These results suggested that there is an induction of enhancement in bSC viability under SMG condition.

Cyclins and cyclin-dependent kinases (Cdks) are fundamental regulators of cell division, driving cell proliferation and playing key roles in controlling cell cycle progression [23]. Cyclins A1 and A2 are particularly important during the S phase and the transition from G2 to M phase. Cyclin D1 acts as a critical regulator of the G1–S transition, linking extracellular signals to the control of cell cycle progression and proliferation [24]. Additionally, the Cyclin A–CDK2 complex forms during the S phase and is essential for the continuation of DNA replication and the subsequent transition into mitosis [25]. In this study, we observed that after 3 days of simulated microgravity (SMG) exposure, the expression levels of key cell cycle regulatory proteins were reduced. This downregulation increased the proportion of cells in the G0/G1 phase, promoting entry into a quiescent state and consequently reducing cell proliferation under SMG conditions. By day 7, however, the expression of cyclin D1 and CDK2 was upregulated, facilitating the transition of cells from the G0/G1 phase to the S phase. These findings suggest that bSCs partially recovered their proliferative capacity and adapted to SMG conditions over time [26]. Nevertheless, cell proliferation in the SMG group remained lower than that of the control group at day 7, indicating that additional mechanisms may be involved and warrant further investigation.

These findings have important implications for cultured meat production using bovine satellite cells (bSCs). Efficient cell expansion is essential for scalable biomass generation; however, SMG-induced suppression of proliferation, G0/G1 arrest, and nuclear remodeling suggest reduced expansion efficiency under altered mechanical conditions. Although increased metabolic activity indicates maintained or adaptive cell viability, the imbalance between proliferation and metabolism may limit overall yield. The partial recovery of cyclin D1 and CDK2 expression at day 7 further suggests an adaptive response, but not sufficient to restore normal growth rates. Therefore, precise control of mechanotransduction cues will be critical to optimize bSC proliferation and ensure efficient, scalable cultured meat production.

## Figures and Tables

**Table 1:** Cell cycle progression analysis.

Cell cycle phase	Day 3		Day 7	
	Control	SMG	Control	SMG
*Sub G1*	8.68 ± 1.62 %	5.65 ± 1.74 %	9.14 ± 1.73 %	2.50 ± 0.96 %
*G0/G1*	73.34 ± 2.13 %	80.51 ± 1.77 %	77.71 ± 3.09 %	76.75 ± 1.04 %
*S*	4.88 ± 1.23 %	2.34 ± 0.39 %	3.64 ± 2.37 %	7.92 ± 1.78 %
*G2/M*	11.62 ± 2.92 %	10.46 ± 1.36 %	8.22 ± 1.91 %	10.73 ± 2.82 %
